# Evaluation of algorithms using administrative health and structured electronic medical record data to determine breast and colorectal cancer recurrence in a Canadian province: Using algorithms to determine breast and colorectal cancer recurrence

**DOI:** 10.1186/s12885-021-08526-9

**Published:** 2021-07-01

**Authors:** Pascal Lambert, Marshall Pitz, Harminder Singh, Kathleen Decker

**Affiliations:** 1 CancerCare Manitoba Research Institute, 675 McDermot Avenue, Winnipeg, Manitoba R3E 0V9 Canada; 2 Department of Epidemiology and Cancer Registry, CancerCare Manitoba, 675 McDermot Avenue, Winnipeg, Manitoba R3E 0V9 Canada; 3 Department of Medical Oncology, CancerCare Manitoba, 675 McDermot Avenue, Winnipeg, Manitoba R3E 0V9 Canada; 4 Department of Internal Medicine, University of Manitoba, 820 Sherbrook Street, Winnipeg, Manitoba R3A 1R9 Canada; 5 Department of Community Health Sciences, University of Manitoba, 750 Bannatyne Avenue, Winnipeg, Manitoba R3E 0W3 Canada

**Keywords:** Breast cancer, Colorectal cancer, Recurrence, Algorithms, Validation studies, Canada

## Abstract

**Background:**

Algorithms that use administrative health and electronic medical record (EMR) data to determine cancer recurrence have the potential to replace chart reviews. This study evaluated algorithms to determine breast and colorectal cancer recurrence in a Canadian province with a universal health care system.

**Methods:**

Individuals diagnosed with stage I-III breast or colorectal cancer diagnosed from 2004 to 2012 in Manitoba, Canada were included. Pre-specified and conditional inference tree algorithms using administrative health and structured EMR data were developed. Sensitivity, specificity, positive predictive value (PPV), negative predictive value (NPV) correct classification, and scaled Brier scores were measured.

**Results:**

The weighted pre-specified variable algorithm for the breast cancer validation cohort (*N* = 1181, 167 recurrences) demonstrated 81.1% sensitivity, 93.2% specificity, 61.4% PPV, 97.4% NPV, 91.8% correct classification, and scaled Brier score of 0.21. The weighted conditional inference tree algorithm demonstrated 68.5% sensitivity, 97.0% specificity, 75.4% PPV, 95.8% NPV, 93.6% correct classification, and scaled Brier score of 0.39. The weighted pre-specified variable algorithm for the colorectal validation cohort (*N* = 693, 136 recurrences) demonstrated 77.7% sensitivity, 92.8% specificity, 70.7% PPV, 94.9% NPV, 90.1% correct classification, and scaled Brier score of 0.33. The conditional inference tree algorithm demonstrated 62.6% sensitivity, 97.8% specificity, 86.4% PPV, 92.2% NPV, 91.4% correct classification, and scaled Brier score of 0.42.

**Conclusions:**

Algorithms developed in this study using administrative health and structured EMR data to determine breast and colorectal cancer recurrence had moderate sensitivity and PPV, high specificity, NPV, and correct classification, but low accuracy. The accuracy is similar to other algorithms developed to classify recurrence only (i.e., distinguished from second primary) and inferior to algorithms that do not make this distinction. The accuracy of algorithms for determining cancer recurrence only must improve before replacing chart reviews.

## Background

Cancer recurrence is the diagnosis of a second clinical episode of cancer after the first was considered cured. It occurs from residual microscopic disease which was not clinically detectable and is different from cancer progression which is due to the growth of known clinical disease. As novel cancer treatments and screening have been introduced and survival has improved, cancer recurrence has become an important outcome; it often results in additional treatment, is a predictor for subsequent mortality, and can be used to compare treatment effectiveness, measure recurrence-free survival, and plan and prioritize cancer control resources [1].

Since 1956, the Manitoba Cancer Registry (MCR) in the province of Manitoba, Canada has been legislated to collect, classify, and maintain population-based information about cancer cases including diagnosis date, histology, topography, stage, and treatment for the entire provincial population. The MCR has been consistently shown to be of very high quality, completeness, and histological verification [2]. Unfortunately, cancer registries, including the MCR, do not systematically identify recurrent cancers. Therefore, recurrence is determined using manual chart review. Manual chart reviews can provide detailed and reflective information. However, they have several important disadvantages. In order to complete a chart review in a timely manner, multiple abstractors are usually required which may introduce error and bias, especially if data abstraction processes are not clear and inter-rater reliability is low. Manually reviewing charts is also labour intensive, time consuming, and, hence, can be expensive.

An alternative method for identifying recurrence is to use existing structured health care data. Several prior studies have developed and validated algorithms for determining cancer recurrence using structured health data in the United States (US), Europe, and Canada [3–10]. Each of these studies has limitations including limited sources of data, narrowly defined populations, different definitions of recurrence, missing validation cohorts, and the use of suboptimal performance measures which do not account for prevalence. Based on the importance of determining recurrence, the inefficiencies of chart reviews, and the limitations of previous recurrence algorithm studies, our goal was to evaluate algorithms to determine recurrence in breast and colorectal cancer cohorts using administrative health and structured electronic medical record (EMR) data in a Canadian province with a universal health care system.

## Methods

### Setting

The province of Manitoba, located in central Canada, has a population of 1.37 million (as of 2019) [11]. Approximately 55% of the population live in the capital city of Winnipeg. Manitoba Health, Seniors and Active Living (MHSAL), the publicly funded provincial health insurance agency, provides comprehensive universal health coverage for hospitalizations, procedures, and physician visits for provincial residents. MHSAL maintains several electronic databases to monitor health care use and reimburse health care providers for services delivered. Since 1984, provincial residents have been assigned a personal health identification number (PHIN) which can be used to link provincial health information databases allowing health care utilization and outcomes to be tracked longitudinally.

### Data sources

The MCR was used to identify individuals diagnosed with breast or colorectal cancer, cancer diagnosis date, age at diagnosis, cancer stage, estrogen receptor/progesterone receptor (ER/PR) and human epidermal growth factor receptor 2 (HER-2) status, date and type of cancer surgery, and date of the first radiation treatment for each course of radiation treatment. The CancerCare Manitoba (CCMB) electronic medical record is the record of clinical cancer interactions, investigations, and treatment, and was used to determine dates and types of systemic anti-cancer medical therapy as well as carcinoembryonic antigen (CEA) and cancer antigen 15–3 (Ca 15–3) blood test results.

We used three MHSAL administrative databases: the Manitoba Population Registry, the Medical Claims database, and the Drug Program Information Network (DPIN) database. The Manitoba Population Registry contains demographic, vital status, and migration information and was used to determine the start and end dates of provincial health coverage. The Medical Claims database is generated by claims filed by health care providers for reimbursement of service and includes services provided, diagnosis, provider, and service date. Medical Claims data were used to determine palliative care consultations. The DPIN database includes all prescriptions dispensed from outpatient pharmacies in Manitoba. DPIN data was used to determine capecitabine, a chemotherapy drug used to treat different cancers including breast and colorectal cancer. Laboratory data were obtained from Shared Health, Manitoba’s public sector laboratory, to identify CEA and Ca15–3 blood test results which were not already in the CCMB medical record. The accuracy and completeness of Manitoba Health’s administrative data has been previously established [12–14].

### Study population

The study included individuals diagnosed with stage I-III colorectal cancer (International Classification of Diseases, Oncology 3rd edition (ICD-O-3) codes C18.0. C18.2–9, C19, C20, C26.0) or breast cancer (ICD-O-3 codes C50.0–6, C50.8–9). Stage IV cases, which have metastasis at diagnosis, were excluded as these individuals develop progression (i.e., worsening disease) rather than recurrence.

The study population was divided into a training cohort of individuals diagnosed from 2004 to 2007 and a validation cohort of individuals diagnosed from 2008 to 2012. Breast and colorectal cancers were analyzed separately. The breast cancer training cohort included cancers that were either ER negative, PR negative, or HER-2 positive because these cancers have a higher recurrence rate and therefore decreased the number of cases needed to review [15]. The colorectal cancer training cohort focused on stage II and III because they are expected to have higher rates of recurrence compared to stage I cancers [16]. The breast and colorectal cancer validation cohorts included individuals diagnosed with stage I-III cancers. However, the breast cancer cohort was oversampled with ER negative, PR negative, and HER-2 cases and the colorectal cancer cohort was oversampled with higher stages to ensure that enough recurrences were identified. The validation cohort included individuals diagnosed in later years to provide external validation, which is a more rigorous validation method than internal or apparent validation [17–19].

### Study variables

Study variables are summarized in Table 1. Cancer recurrence included loco-regional (reappearance of cancer in the same region of the body or the lymph nodes) and distant (reappearance of cancer in another part of the body) recurrence. Surgery and radiation treatment data were linked by a tumour ID which identifies the treatment associated with a specific tumour. Therefore, if an individual had more than one cancer diagnosis, the treatment data could be linked to the appropriate cancer. The remaining variables could not be linked to a specific tumour. To increase accuracy in classifying the remaining variables, conditions were added. Surgery (mastectomy, lumpectomy, axillary lymph node dissection for breast cancer and bypass or resection surgery for CRC) beyond 12 months after diagnosis were included to capture local recurrence after the use of neoadjuvant treatments. New disease within 1 year is usually considered part of the primary diagnosis. Surgery for a non-breast site or liver or lung resections beyond 6 months were included to capture treatment for metastases after the initial treatment. The receipt of chemotherapy beyond 12 months of diagnosis and RT beyond 12 months for breast cancer were considered due to recurrence unless the treatments occurred after a second primary treated with surgery. Although chemotherapy treatment could not be linked to a specific tumour, the treatment site was identified which increased accuracy of correct association. A palliative care consult was considered due to recurrence if it was provided by an oncologist and was linked to a breast cancer (breast cancer cases only), colorectal cancer (colorectal cancer cases only), lung cancer, liver cancer, or undetermined metastases beyond 6 months after diagnosis to exclude any treatment discussions that may have occurred after diagnosis. Elevated blood markers (CEA > 10; Ca 15–3 > 50) often related to recurrence more than 12 months after diagnosis were considered due to recurrence unless they occurred within 3 months of another primary cancer diagnosis. Elevated blood markers prior to 12 months were not included to avoid initial elevations due to the original diagnosis or treatment.

**Table 1:** Variables included in the study

Variable	Data source	Time frame after diagnosis
**Breast Cancer**		
Surgery (mastectomy, lumpectomy, axillary lymph node dissection)	Manitoba Cancer Registry	12 months
Surgery (non-breast site)	Manitoba Cancer Registry	6 months
Chemotherapy	CancerCare Manitoba EMR^c^ Drug Program Information Network	12 months^a^
Radiation therapy	Manitoba Cancer Registry	12 months
Palliative care consultation from oncologist	Medical Claims	6 months
CEA > 10	CancerCare Manitoba EMR Shared Health	12 months^b^
Ca 15–3 > 50	CancerCare Manitoba EMR Shared Health	12 months^b^
**Colorectal cancer**		
Surgery (bypass or resection)	Manitoba Cancer Registry	12 months
Surgery (liver or lung resection)	Manitoba Cancer Registry	6 months
Chemotherapy	CancerCare Manitoba EMR Drug Program Information Network	12 months^a^
Palliative care consultation from oncologist	Medical Claims	6 months
CEA > 10	CancerCare Manitoba EMR Shared Health	12 months^b^

^a^Excluding individuals who had chemotherapy after a second primary cancer diagnosis treated with surgery.

^b^Excluding individuals who had elevated blood markers within 3 months of another primary cancer diagnosis.

^c^Electronic medical record

### Algorithm development and validation

A chart review was first conducted by trained research assistants to identify cancer recurrence. A duplicate chart review by a research assistant who did not conduct the initial chart review was conducted for a fraction of the cohort (10%) to evaluate inter-rater reliability. The algorithms were then developed by analyzing the same cohorts using two approaches: pre-specified variables and conditional inference trees. The pre-specified variable approach used variables and clinically meaningful cut offs determined prior to the start of the study. Variables and cut-offs were selected with information from previous studies and local cancer experts. For this algorithm, if an individual was positive for any of the variables included, they were predicted to have a recurrence. The conditional inference tree approach is an automated machine learning technique that explicitly states the algorithm that was developed, which is not achieved with other machine learning techniques. The conditional inference trees used the same variables as the pre-defined algorithm. However, trees were created based on the association between each covariate and the outcome of interest (i.e., recurrence). The *ctree* function within the party R package with a default setting (a quadratic test statistic, Bonferroni-adjusted *p*-values, and criterion p-value of 0.05) [20]. Validation cohorts were used to determine if the algorithms developed were generalizable to cancer cohorts independent of those analyzed as part of the algorithm development.

### Performance metrics

Sensitivity (the percentage of individuals who had a recurrence that were correctly identified), specificity (the percentage of individuals who did not have a recurrence that were correctly identified), positive predictive value (PPV) (percentage of individuals predicted to have recurrence that truly have recurrence), negative predictive value (NPV) (percentage of individuals predicted to not have recurrence that truly do not have recurrence), correct classification (the percentage of individuals who were correctly classified as having a recurrence or not having a recurrence), and scaled Brier scores were calculated to determine algorithm accuracy. These classification measures are commonly used in the literature to describe the performance of algorithms and models. Brier scores measure the predictive accuracy by subtracting the predictive values from the outcome values (the average of squared differences between predicted values and outcome values). The Brier score was then scaled to the proportion of events (p) in the cohort 1-(Brier score/(mean(p)*(1-mean(p)))) where a value of 1 is perfect prediction, a value of 0 is chance, and a negative value is worse than chance) [21, 22]. Therefore, unlike measures like sensitivity, specificity, and correct classification, the scaled Brier score is adjusted for the prevalence of events in the cohort. This is advantageous in measuring accuracy over commonly used classification measures which ignore prevalence. Measures were unweighted for both training and validation cohorts. Weighted measures were also calculated for the validation cohort to account for oversampling. Confidence intervals were determined using the permutation method, including 1000 replications and reporting values at the 2.5th and 97.5th percentiles.

## Results

### Breast cancer

The breast cancer training cohort included 933 cases with 186 recurrences and the validation cohort included 1181 cases with 167 recurrences (Table 2). The mean age at diagnosis was 60.3 years (standard deviation (SD) 14.1) in the training cohort and 62.5 years (SD 13.9) in the validation cohort. In the training cohort, 36.2% were diagnosed at stage I, 45.7% at stage II, and 18.1% at stage III. The stage distribution was similar in the validation cohort. Two-hundred and seven charts were reviewed by a second research assistant and the Kappa statistic for recurrence status was 0.81.

**Table 2:** Characteristics of individuals diagnosed with breast or colorectal cancer in the training and validation cohorts

	Breast cancer, N, (%)		Colorectal cancer, N, (%)	
	Training cohort (*N* = 933)	Validation cohort (*N* = 1811)	Training cohort (*N* = 620)	Validation cohort (*N* = 693)
**Age at diagnosis (mean, (SD))**	60.3 (14.1)	62.5 (13.9)	69.4 (12.6)	67.9 (12.3)
**Stage**				
**I**	338 (36.2)	487 (41.2)	0 (0)	138 (19.9)
**II**	426 (45.7)	485 (41.1)	285 (46.0)	236 (34.1)
**III**	169 (18.1)	209 (17.7)	335 (54.0)	319 (46.0)
**Recurrences**	186 (19.9)	167 (9.2)	126 (20.3)	136 (19.6)

**Fig. 1:**
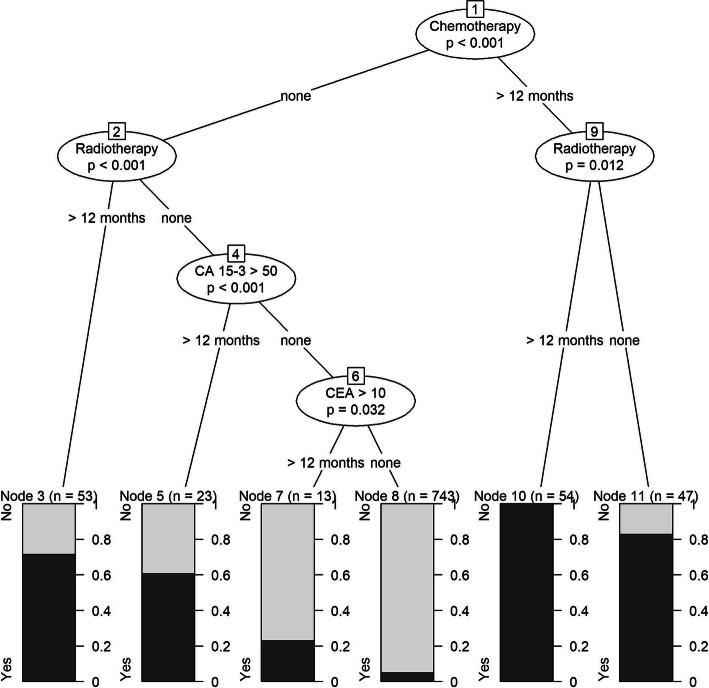
Conditional inference tree for breast cancer recurrence. Legend: CA-15-3, cancer antigen 15–3 blood test results; CEA, carcinoembryonic antigen blood test; Yes, recurrence; No, no recurrence

Table 3 shows the performance metrics for determining breast cancer recurrence for the training and validation cohorts, unweighted and weighted, using pre-specified variable and conditional tree algorithms. In the validation cohort, the pre-specified variable algorithm demonstrated the following weighted results: 81.1% sensitivity, 93.2% specificity, 61.4% PPV, 97.4% NPV, and 91.8% correct classification. The weighted scaled Brier score was 0.21 which demonstrates low accuracy. The conditional inference tree algorithm for the breast cancer training cohort is shown in Fig. 1. The percentage of individuals who were classified as having had a recurrence are shown for each node in the tree. For example, 70% of individuals who did not have chemotherapy but had radiation therapy greater than 12 months after diagnosis were classified as having had a recurrence (node 3, *N* = 53). Overall, the conditional inference tree algorithm demonstrated the following weighted results: 68.5% sensitivity, 97.0% specificity, 75.4% PPV, 95.8% NPV, and 93.6% correct classification. The weighted scaled Brier score was 0.39.

**Table 3:** Performance metrics for determining breast cancer recurrence using pre-specified variable algorithm and conditional tree algorithms

	Pre-defined variable algorithm, % (95% CI^c^)			Conditional tree algorithm, % (95% CI)		
	Training cohort (*N* = 933)	Validation cohort (*N* = 1811)		Training cohort (*N* = 933)	Validation cohort (*N* = 1811)	
		Unweighted	Weighted		Unweighted	Weighted
**Sensitivity**	83.3 (77.9–88.2)	83.2 (77.2–88.6)	81.1 (77.4–84.8)	78.0 (72.0–83.9)	73.7 (67.1–79.7)	68.5 (64.6–72.4)
**Specificity**	89.7 (85.0–93.6)	92.5 (88.1–95.8)	93.2 (90.6–95.4)	95.7 (92.5–98.4)	96.4 (93.4–98.8)	97.0 (95.4–98.5)
**PPV**^a^	66.8 (57.9–76.7)	64.7 (54.0–76.8)	61.4 (53.7–70.2)	81.9 (72.0–92.4)	73.7 (63.5–91.1)	75.4 (66.3–85.7)
**NPV**^b^	96.0 (94.2–96.9)	97.1 (96.1–98.0)	97.4 (96.8–97.9)	94.6 (93.2–96.0)	96.4 (94.7–96.7)	95.8 (95.3–96.3)
**Correct classification**	88.4 (84.5–91.9)	91.1 (87.6–94.2)	91.8 (89.4–93.8)	92.2 (89.5–94.6)	93.1 (90.3–95.6)	93.6 (92.1–94.9)
**Scaled Brier**	0.27 (0.03–0.49)	0.27 (−0.03–0.53)	0.21 (−0.01–0.40)	0.51 (34.2–66.4)	0.44 (0.20–0.64)	0.39 (0.24–0.51)

^a^Positive predictive value

^b^Negative predictive value

^c^Confidence interval

### Colorectal cancer

The colorectal cancer training cohort included 620 cases with 126 recurrences and the colorectal cancer validation cohort included 693 cases with 136 recurrences (Table 2). The mean age at diagnosis was 69.4 years (SD 12.6) in the training cohort and 67.9 years (SD 12.3) in the validation cohort. In the training cohort, no individuals were included who were diagnosed at stage I; 46.0% were diagnosed at stage II and 54.0% were diagnosed at stage II. In the validation cohort, 19.9, 34.1, and 46.0% were diagnosed at stages I, II, and III, respectively. One-hundred and twenty-eight charts were reviewed by a second research assistant and the Kappa statistic for recurrence status was 0.73.

**Fig. 2:**
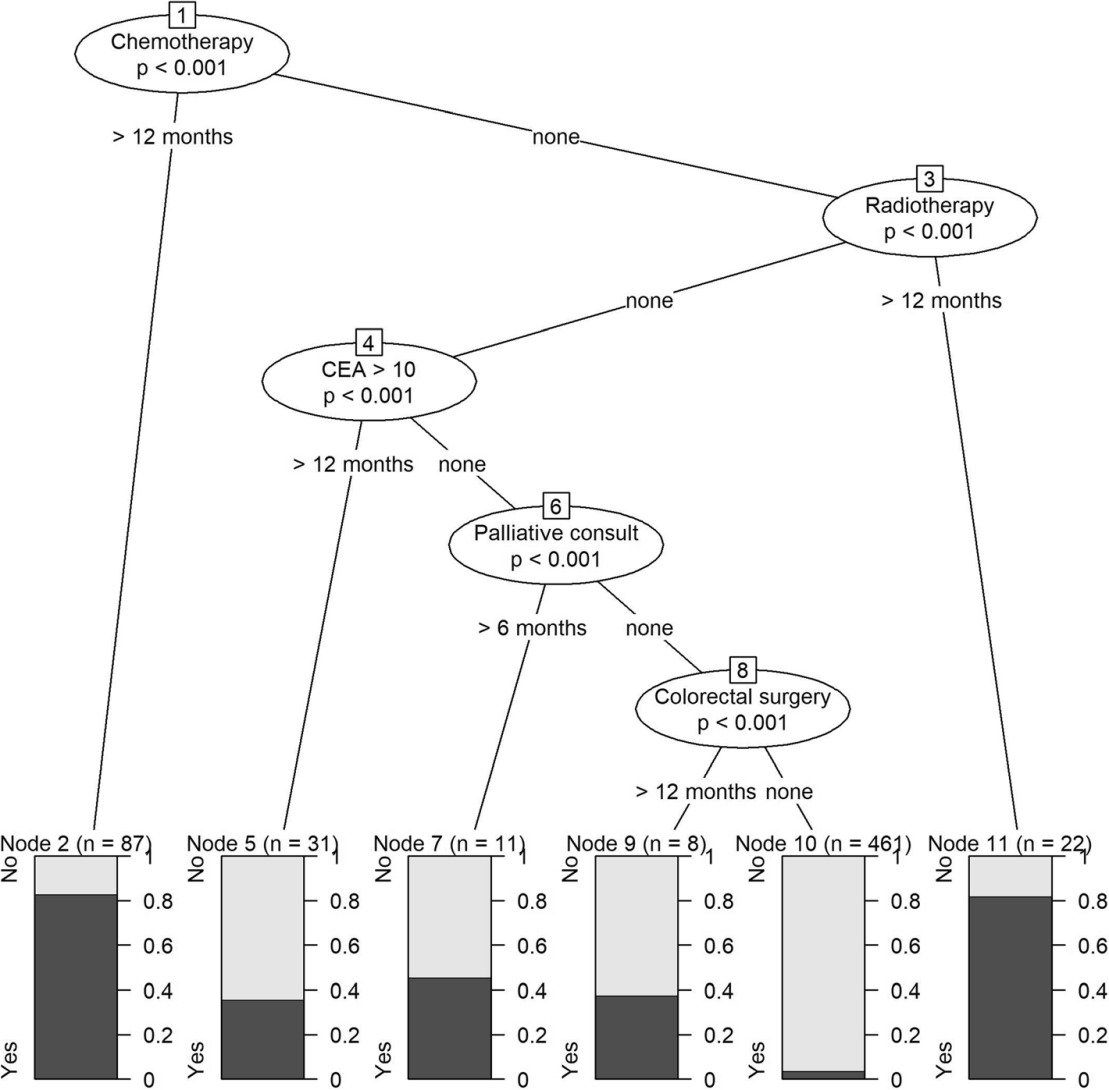
Conditional inference tree for colorectal cancer recurrence. Legend: CEA, carcinoembryonic antigen blood test; Yes, recurrence; No, no recurrence

Table 4 shows the performance metrics for determining colorectal cancer recurrence for the training and validation cohorts, both unweighted and weighted, using the pre-specified variable and conditional tree algorithms. In the validation cohort, the pre-specified variable algorithm demonstrated the following weighted results: 77.7% sensitivity, 92.8% specificity, 70.7% PPV, 94.9% NPV, and 90.1% correct classification. The weighted scaled Brier score was 0.33. The conditional inference tree algorithm developed demonstrated the following weighted results: 62.6% sensitivity, 97.8% specificity, 86.4% PPV, 92.2% NPV, and 91.4% correct classification (Fig. 2). The weighted scaled Brier score was 0.42.

**Table 4:** Performance metrics for determining colorectal cancer recurrence using pre-defined variable algorithms and conditional inference tree algorithms

	Pre-defined variable algorithm, % (95% CI^c^)			Conditional tree algorithm, % (95% CI)		
	Training cohort (*N* = 933)	Validation cohort (*N* = 1811)		Training cohort (*N* = 620)	Validation cohort (*N* = 693)	
		Unweighted	Weighted		Unweighted	Weighted
**Sensitivity**	88.1 (81.7–92.9)	79.4 (72.8–86.0)	77.7 (74.1–80.7)	71.4 (63.5–79.4)	64.7 (56.6–72.8)	62.6 (58.9–66.4)
**Specificity**	89.9 (84.2–95.1)	92.5 (87.4–96.4)	92.8 (90.6–94.7)	96.2 (92.1–99.2)	96.9 (93.4–99.3)	97.8 (96.4–98.8)
**PPV**^a^	68.9 (59.0–81.8)	72.0 (60.2–85.1)	70.7 (64.8–76.7)	82.6 (70.3–95.7)	83.8 (71.0–95.9)	86.4 (79.8–92.3)
**NPV**^b^	96.7 (95.1–98.1)	94.8 (93.2–96.4)	94.9 (94.1–95.6)	93.0 (91.2–94.8)	91.4 (90.1–93.6)	92.2 (91.4–92.9)
**Correct classification**	89.5 (84.8–93.7)	89.9 (85.6–93.5)	90.1 (88.1–91.8)	91.1 (87.9–94.0)	90.6 (87.6–93.4)	91.4 (90.1–92.5)
**Scaled Brier**	0.35 (0.06–0.61)	0.36 (0.09–0.59)	0.33 (0.20–0.45)	0.45 (0.25–0.63)	0.41 (0.21–0.58)	0.42 (0.34–0.50)

^a^Positive predictive value

^b^Negative predictive value

^c^Confidence interval

## Discussion

### Main findings

We found that algorithms using administrative health and structured EMR data to determine breast and colorectal cancer recurrence had high to moderate sensitivity and PPV, high specificity, NPV, and correct classification but low accuracy after adjusting for the prevalence of the outcome in the cohort. As expected, training cohort results were higher than validation cohort results because the algorithms were optimized on the training cohorts. We chose to include breast and colorectal cancers as these sites have relatively high survival rates, are the second and third most commonly diagnosed cancers in Manitoba (which makes chart reviews even more costly and time-consuming), and are historically more likely than aggressive cancers with poorer survival to have recurrences that can be effectively treated. Whether or not these algorithms can replace chart reviews for determining cancer recurrence necessitates weighing the costs required to conduct a chart review with the benefit of quickly applying an algorithm with less than optimal accuracy.

### Comparison with other studies

Prior studies that evaluated cancer recurrence algorithms using structured data found moderate to high sensitivities and specificities but have several important limitations. Lamont et al. [3] used Medicare claims data to measure disease-free survival in individuals ≥65 years of age diagnosed with breast cancer (*N* = 45, 12 recurrences and 2 deaths) . Algorithm sensitivity and specificity were 83 and 97% respectively. Rasmussen et al. [6] used national data in Denmark to identify breast cancer recurrence (*n* = 471, 149 recurrences). Sensitivity was 97%, specificity was 97%, and PPV was 94%. Xu et al. [9] developed algorithms to identify breast cancer recurrence among women ≤40 years of age or those who received neoadjuvant chemotherapy in Alberta (*N* = 598, 121 recurrences) . Sensitivity values ranged from 75 to 94% and specificity values ranged from 94 to 98%. Chubak et al. [5] developed several algorithms to determine second breast cancer events and recurrence only among women diagnosed with stage I or II breast cancer (*n* = 3152, 407  breast cancer events). Sensitivity values ranged from 69 to 99% and specificity values ranged from 81 to 99%. These four studies generally demonstrated better accuracy compared to our study. However, one study included a single small training cohort [3] and two studies did not distinguish between recurrence and second breast cancer primary (i.e., a new primary cancer unrelated to the prior cancer) [6, 9]. This distinction is important in order to use the algorithms to evaluate outcomes such as the effectiveness of treatments in preventing a cancer recurrence. We attempted to distinguish recurrence from a second primary in our chart reviews, although this was difficult in some cases. In addition, one study’s results were based on a training cohort which would have produced overly optimistic results [23] and another excluded patients with second primary non-breast tumours [9] which may have introduced bias.

Chubak et al. [5] noted that their algorithms to classify only recurrence generally demonstrated accuracy that were not as high as their algorithms to classify second breast cancer events . Several recurrence-only studies have generally demonstrated lower accuracy in comparison to the previously mentioned studies, as well as similar accuracy to our study results. A recent study (2016) developed a medical claims-based algorithm to identify ovarian cancer recurrence (*N* = 94, 32 recurrences) [4]. Sensitivity was 100% and specificity was 89% but only a training cohort was assessed and the cohort size was small. A large US study (2014) evaluated multiple recurrence algorithms for each cancer site of lung, colorectal, breast, and prostate (*N* = 6227, 736 recurrences) [7]. Sensitivity ranged from 6 to 85% and specificity ranged from 70 to 97%. In 2017, the study was extended to include additional data to be used in the lung and colorectal cancer algorithms: sensitivity ranged from 72 to 91% and specificity ranged from 86 to 98% [8]. Cairncross et al. [10] randomly selected 200 women (26 recurrences) diagnosed with cancer and who had ever had a pregnancy between 2003 and 2012. Sensitivity was 81%, specificity was 81%, PPV was 39%, and NPV was 97%.

Importantly, none of the prior studies used metrics that are optimal to measure algorithm performance such as the scaled Brier score. Sensitivity and specificity are useful because they provide context about how an algorithm can be improved by identifying areas of weakness. For example, some recurrences in our study were missed because the individual did not receive treatment, which decreased sensitivity. This could have been improved if cause of death data were available for the study period. In addition, chemotherapy for a second primary was often found among false positives, which decreased specificity. This could have been improved if chemotherapy data could have been linked to individual tumours rather than to only individuals. However, sensitivity and specificity ignore the rate of events in a cohort which make assessing the overall performance of an algorithm challenging. For example, a specificity or correct classification of 95% will have a high rate of false positives if the rate of event is low (e.g., 1%) but would be substantially better with a higher rate of events (e.g., 50%). The scaled Brier score, which is a summary measure that accounts for the rate of events in a cohort, does not have this limitation. Moreover, if a proposed algorithm is expected to replace a chart review, metrics of accuracy should also indicate the amount of measurement error involved. The scaled Brier score, which has a similar interpretation to the R^2^, indicates random association with a value of 0 and perfect prediction with a value of 1. This provides more informative output to describe accuracy than measures that only use subsections of the cohort (e.g., sensitivity and specificity).

Other methods, such as those that use natural language processing (NLP) to capture recurrence from unstructured EMR data, have been used to determine breast cancer recurrence with sensitivities from that range from 83 to 92% [24–27]. These results are not very different that those that used structured administrative data and therefore, may also not be accurate enough at this time to replace a chart review. Another option is to use recurrence algorithms as a screening tool to reduce the number of charts that need to be manually reviewed. However, more research is needed to create algorithms with higher sensitivities to evaluate this possibility.

### Strengths and limitations

We used data from previously validated, high-quality, complete, population-based administrative health databases [12, 13, 28, 29]. However, our gold standard was a chart review which is subject to human error. The inter-rater reliability was strong for breast cancer and moderate for colorectal cancer [30]. Therefore, there was some disagreement among the chart reviewers about what constitutes recurrence. When investigating samples of false positives and negatives in the training cohort, some misclassifications of recurrence status had occurred. This was often due to the difficulty in distinguishing recurrence from a second primary, which is expected because it is sometimes challenging for physicians to definitively make this determination. We also found that additional chemotherapy may have been due to a second cancer primary and not cancer recurrence leading to false positive cases. Like some prior studies, our definition of recurrence was not time dependent. We chose to not include this because this would only lead to poorer results.

## Conclusions

Our algorithms that used structured administrative health and EMR data to determine recurrence in breast and colorectal cancer cohorts had moderate sensitivity and PPV, high specificity, NPV, and correct classification but low overall accuracy. These results and a review of other studies suggest that more accurate algorithms to capture recurrence-only events are required to replace chart reviews.

## Data Availability

The data that support the findings of this study are not publicly available to ensure and maintain the privacy and confidentiality of individuals’ health information. Requests for may be made to the appropriate data stewards (Manitoba Health, Seniors and Active Living’s Health Information Privacy Committee and CancerCare Manitoba’s Research and Resource Impact Committee).

## Abbreviations

CCMB: CancerCare Manitoba
CEA: Carcinoembryonic antigen
DPIN: Drug Program Information Network
EMR: Electronic medical record
ER/PR: Estrogen receptor/progesterone receptor
HER-2: Human epidermal growth factor receptor 2
ICD-0-3: International Classification of Diseases, Oncology 3rd edition
MCR: Manitoba Cancer Registry
MHSAL: Manitoba Health Seniors and Active Living
NPV: Negative predictive value
PHIN: Personal Health Information Number
PPV: Positive predictive value
SD: Standard deviation
US: United States
